# Crystal structure of MytiLec, a galactose-binding lectin from the mussel *Mytilus galloprovincialis* with cytotoxicity against certain cancer cell types

**DOI:** 10.1038/srep28344

**Published:** 2016-06-20

**Authors:** Daiki Terada, Fumihiro Kawai, Hiroki Noguchi, Satoru Unzai, Imtiaj Hasan, Yuki Fujii, Sam-Yong Park, Yasuhiro Ozeki, Jeremy R. H. Tame

**Affiliations:** 1 Graduate School of Medical Life Science, Yokohama City University, 1-7-29 Suehiro, Yokohama, Kanagawa 230-0045, Japan; 2 Laboratory of Glycobiology and Marine Biochemistry, Graduate School of NanoBio Sciences, Yokohama City University, 22-2, Seto, Yokohama, Kanagawa 236-0027, Japan; 3 Department of Biochemistry and Molecular Biology, Faculty of Science, University of Rajshahi, Rajshahi-6205, Bangladesh; 4 Department of Pharmacy, Graduate School of Pharmaceutical Science, Nagasaki International University, 2825-7 Huis Ten Bosch, Sasebo, Nagasaki 859-3298, Japan

## Abstract

MytiLec is a lectin, isolated from bivalves, with cytotoxic activity against cancer cell lines that express globotriaosyl ceramide, Gal*α*(1,4)Gal*β*(1,4)Glc*α*1-Cer, on the cell surface. Functional analysis shows that the protein binds to the disaccharide melibiose, Gal*α*(1,6)Glc, and the trisaccharide globotriose, Gal*α*(1,4)Gal*β*(1,4)Glc. Recombinant MytiLec expressed in bacteria showed the same haemagglutinating and cytotoxic activity against Burkitt’s lymphoma (Raji) cells as the native form. The crystal structure has been determined to atomic resolution, in the presence and absence of ligands, showing the protein to be a member of the *β*-trefoil family, but with a mode of ligand binding unique to a small group of related trefoil lectins. Each of the three pseudo-equivalent binding sites within the monomer shows ligand binding, and the protein forms a tight dimer in solution. An engineered monomer mutant lost all cytotoxic activity against Raji cells, but retained some haemagglutination activity, showing that the quaternary structure of the protein is important for its cellular effects.

Lectins are non-catalytic glycan-binding proteins, present in all three domains of life, with diverse stereospecific carbohydrate binding specificities, functions and protein folds. MytiLec, a lectin isolated from the Mediterranean mussel *Mytilus galloprovincialis*, was identified through a study of marine organisms and their adaptation to different habitats and environmental systems, and shown to bind *α*-D-galactose and N-acetyl-*α*-D-galactosamine (*α*-GalNAc)^1^. The primary structure of MytiLec, with 149 amino acid residues in total (http://www.uniprot.org/uniprot/B3EWR1), was determined by a protein chemical procedure combining both Edman degradation and mass spectrometry. It has three broadly similar repeats of a sequence about 45 residues long, highly conserved at certain positions, and a single tryptophan residue near the start of the second repeat. MytiLec has no significant sequence similarity to any known protein, except *Crenomytilus grayanus* lectin (CGL)^2^ and *Mytilus trossulus* lectin (MTL)^3^, from two different species of sea mussel, which both share 88% sequence identity with MytiLec. The natural function of these proteins appears to be anti-bacterial or anti-fungal, and they show bacteriostatic properties. Lectins from *Mytilus galloprovincialis* are known to be involved in innate immunity by acting as pattern recognition receptors (PRR)^4^,^5^. Recently genetics approaches have identified two new related proteins in *M*. *galloprovincialis* named MytiLec-2 and MytiLec-3, which consist of both a carbohydrate-binding domain with a very similar primary structure to MytiLec and a pore-forming aerolysin-like domain^6^. We have shown^1^ that MytiLec binds globotriose (abbreviated Gb3), Gal*α*(1,4)Gal*β*(1,4)Glc, a component of glycosphingolipids that is prevalent on the surface of certain cancer cell types such as Burkitt’s lymphoma^7^. MytiLec added to such cells down-regulates cell growth and shows dose-dependent cytotoxic effects that are specifically blocked by the addition of an *α*-galactoside^1^.

Recently there has been a huge increase in interest in the medicinal potential of lectins, particularly as treatments for cancer^8^, but also as adjuvants or modulators of immune responses^9^, and anti-viral agents^10^. Exogenous expression of some lectins derived from marine sources has been shown to induce apoptosis in cancer cells^11^, but the marked cytotoxicity of many lectins substantially reduces the possibility of developing them into clinically useful formulations. The lectin ABL, from the edible mushroom *Agaricus bisporus*, combines both strong antiproliferative effects on epithelial cancer cells with no apparent cytotoxicity to normal cells^12^,^13^. It recognises the Thomsen-Friedenreich-antigen (TF-antigen), a well-known disaccharide cell-surface marker for neoplastic cells, consisting of a galactose and N-acetyl-D-galactosamine residue connected by a *β*(1,3) linkage. After uptake into human colon cancer cells, ABL appears to block nuclear localization signal-dependent protein uptake into the nucleus^14^. A related lectin has also been isolated from another common edible mushroom, *Boletus edulis*, and named BEL *β*-trefoil^15^. The majority of lectins such as ABL and BEL *β*-trefoil that have been studied for potential medical benefits are specific for *β*-linked saccharides, so MytiLec is of interest for its unusual ligand specificity and anti-bacterial properties, as well as its demonstrated effects against certain cancer cell lines.

Although relatively rare, *β*-trefoil lectins with a specificity for *α*-linked oliogosaccharides have been structurally characterised, including the agglutinin MOA from the fairy ring mushroom *Marasmius oreades*, which is specific for Gal*α*(1,3) Gal-containing saccharides, and can agglutinate group B erythrocytes^16^,^17^. The *β*-trefoil domain of MOA is also slightly unusual in having three sugar binding sites related by pseudo-symmetry. To understand the ligand specificity and 3-dimensional structure of MytiLec, we have created a bacterial expression system for the protein, and solved its structure with crystals diffracting to atomic resolution. Lack of a suitable search model with high sequence similarity to MytiLec led us to determine phases experimentally. The conformation of a complex formed by MytiLec with *α*-GalNAc was also solved, in a different space-group from the apo form.

## Results

### Overall structure

**Figure 1:**
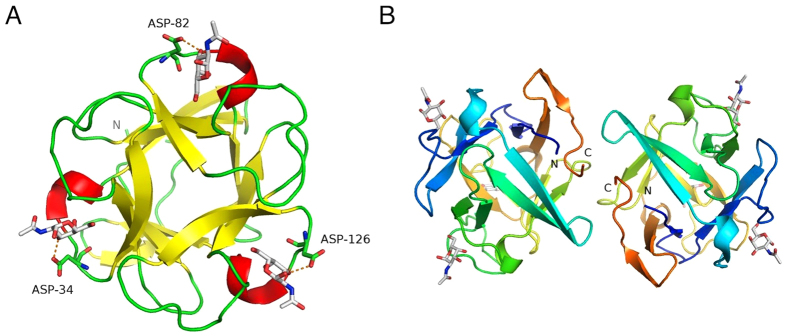
The overall structure of MytiLec. (**A**) The C*α* trace of one MytiLec subunit, looking along the pseudo-three-fold symmetry axis. The trace is coloured by secondary structure, with *α*-helices shown as red coils and *β*-strands as yellow arrows. *α*-GalNAc ligands are shown as sticks with white carbon atoms, and the conserved aspartic acid residue at each site is shown as sticks and labelled. The hydrogen bond between this aspartate and the ligand is shown as a dashed line. Secondary structure was determined automatically by PYMOL^46^. (**B**) The MytiLec dimer, viewed down the dyad axis. Each subunit is coloured from blue (N-terminus) to orange (C-terminus). *α*-GalNAc ligands are shown as sticks, with carbon atoms white and oxygen atoms red.

**Figure 2:**
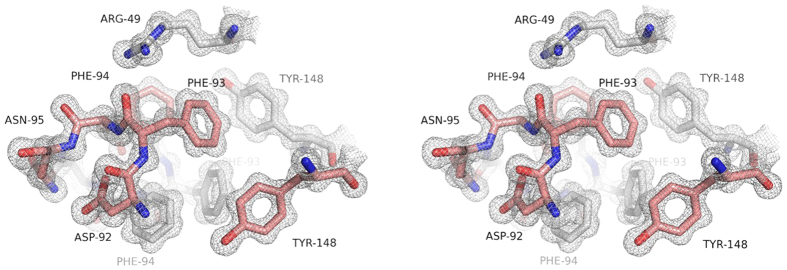
Stereo view of the 2mFo-DFc electron density map in the region of the subunit interface of apo-MytiLec. Carbon atoms are shown in brown for one subunit and white for the other. Oxygen atoms are coloured red and nitrogen blue. Residues Phe 93 and Phe 94 from each subunit contribute significantly to the apolar interface. The map is contoured at 1 *σ*.

**Figure 3:**
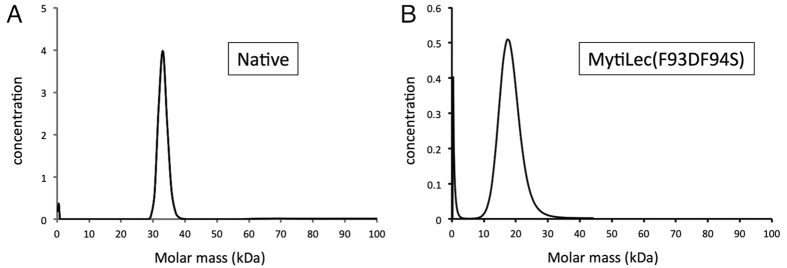
Sedimentation velocity analytical ultracentrifugation analysis of apo-MytiLec. (**A**) The native protein showed a molecular weight of 34 kDa, indicating a dimer is present in solution, with negligible contribution to the sedimentation curves from a component corresponding to the monomer. The concentration, *c*(*M*), is measured in absorption units (A280). (**B**) Sedimentation of MytiLec(F93DF94S), showing the mutant protein adopts a monomeric form with no dimer present. The monomer form was relatively insoluble compared to native protein, necessitating the use of much lower concentration.

Apo-MytiLec crystallised in space-group *P*2_1_, and diffracted to 1.1 Å resolution. The lack of an obvious search model led us to determine phases using a heavy atom derivative, which was aided by the strong diffraction of the physically robust crystals. Crystallographic data and refinement statistics are given in Table 1. Two monomers of MytiLec are found in the asymmetric unit, and the model shows expected geometrical features and has no Ramachandran outliers. Comparison of the two partner chains shows no large shifts of equivalent residues in the Ramachandran plot, although several residues show alternative side-chain conformations. The structure is well ordered, with the entire main-chain of each monomer visible in the 2mFo-DFc electron density map, revealing a *β*-trefoil fold consisting of three related sub-domains (Fig. 1). Several N-terminal residues remaining from the thrombin-cleavable tag are also present in the apo-MytiLec electron density map, so that chain numbering of the apo structure starts at -1 or -3. Each sub-domain of the polypeptide chain essentially consists of a four-stranded *β*-sheet, the last sub-domain having one extra strand at the C-terminus of the protein, giving 13 strands in total per chain. Least-squares fitting the C*α* atoms of residues 1 to 149 of each chain in the apo-MytiLec model gives a rmsd of 0.27 Å. Overlaying 37 central C*α* atoms of individual sub-domains from within the same chain gives rmsd values no higher than 0.6 Å, indicating a high degree of internal symmetry (Supplementary Fig. S1). The two monomers in the asymmetric unit are closely associated by hydrophobic interactions and hydrogen bonds, as shown in Supplementary Tables S1 and S2. PISA^18^ reports the buried interface area between MytiLec monomers to be about 740 Å^2^ per chain. Two adjacent phenylalanine residues, Phe 93 and Phe 94, sit opposite their symmetry mates at the dimer interface (Fig. 2). MytiLec was originally reported to be a monomer in solution on the basis of gel filtration^1^ but analytical ultracentrifugation confirms the protein is a tightly-bound dimer, and mutation of Phe 93 and Phe 94 blocks dimerisation (Fig. 3).

**Figure 4:**
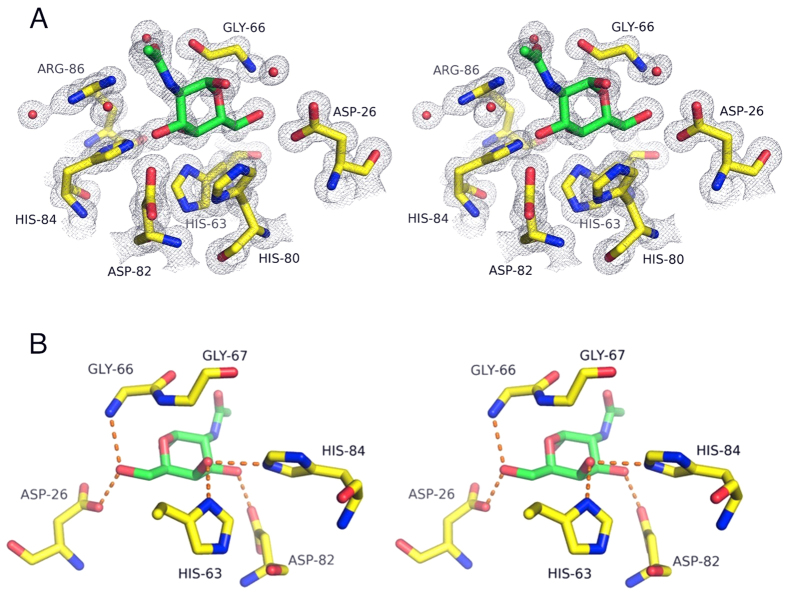
Interactions between MytiLec and GalNAc. (**A**) A stereo figure of the 2mFo-DFc electron density map for the refined complex model, covering the molecule of *α*-GalNAc in binding site 2. Electron density is shown at a level of 1 *σ*. Carbon atoms are shown in yellow for the protein and green for the ligand. Oxygen atoms are coloured red and nitrogen blue. The maps shows clear rings even for the histidine side-chains. (**B**) A different view of the same bound ligand as in (**A**), and with hydrogen bonds shown as orange dotted lines. The acetyl group points away from the protein and makes no contact with it. At the binding site shown, His 63 and His 84 form hydrogen bonds to the epimeric oxygen O4, ensuring selectivity for galactose over glucose. Asp 26 of binding site 2 is equivalent to Glu 118 and Glu 74 in binding sites 1 and 3 respectively, and each makes a hydrogen bond with O6.

In order to understand how MytiLec binds to galactose and GalNAc, and whether the acetyl group contacts the protein, co-crystals of MytiLec and GalNAc were grown. These crystals, in space-group *C*2, diffracted to slightly higher resolution than the apo crystals (Table 1), and also had a dimer in the asymmetric unit showing identical interactions between partner chains. Six GalNAc molecules were identified in the electron density map, and were sufficiently ordered for the electron density map to show a large hole in the centre of each ring (Fig. 4A).

### Comparison with other trefoil lectins

MytiLec shows very strong sequence similarity to CGL, and the crystal structure of apo-CGL was reported in 2015^19^. This model (PDB 5DUY) was refined to 2.12 Å resolution, was solved by molecular replacement using a combination of 39 known trefoil models. MytiLec and CGL show an essentially identical protein fold, and Jakob and colleagues used our MytiLec structures to model sugar binding to CGL^19^. More recently, higher resolution models of CGL have been described with several different ligands bound including galactosamine and Gb3 allyl^20^. A sequence alignment of MytiLec and CGL made with ENDscript^21^ showing the structural similarity is given in Supplementary Fig. S2. Similar trefoil structures were identified in PDB using DALI^22^. The closest match is CGL with a Z-score of 33.3, but the sequence similarity of all other protein hits was under 12% overall. The next hit, with Z = 21.2, was an exo-beta-1,3-galactanase from *Clostridium thermocellum* (PDB 3VT1), but the models of this protein are refined to only moderate resolution^23^. Threefoil, an artificial symmetrical *β*-trefoil was the next best structural match, with Z = 20.8^24^. Agglutinin-II from the bark of the elderberry *Sambucas nigra* is the next highest score (Z = 19.1); it binds the Tn antigen (GalNAc linked to serine or threonine), but is mainly monomeric in solution^25^. The MOA N-terminal trefoil domain shows strong similarity to MytiLec (Z = 18.6), but the dimerisation is through the C-terminal domain^16^. Several dimeric *β*-trefoil lectins are known that, like MytiLec, have no other domains and self-associate by direct interaction of the trefoil. Two such proteins, SSA, an agglutinin from the phytopathogenic fungus *Sclerotinia sclerotiorum* (PDB 2X2T)^26^, and BEL *β*-trefoil (PDB 4I4X)^15^ were chosen for comparison of the dimerisation interface. Overlaps of the monomers were made using secondary-structure matching^27^. For MytiLec-SSA, 120 residues were fitted with 12.5% sequence identity and a rmsd of 1.5 Å. For MytiLec-BEL *β*-trefoil, this fitted 106 residues with about 10% sequence identity, and gave a rmsd of 1.7 Å for the C*α* atoms. The three proteins are of similar length and show highly related topology, the *β*-strands sitting in closely related positions. The surface structure is substantially different however, and the loops are not conserved. Overlaying the models, it can be seen the sugar-binding sites are at equivalent topological positions, but the manner of sugar binding is very different, and the residues found at the binding sites of MytiLec (described below) are not preserved. SSA shows strong sugar-binding at only one site per monomer^26^, which overlaps a binding site on MytiLec. MytiLec, BEL *β*-trefoil and SSA have a very different manner of dimerisation, so that overlaying a monomer of each protein leaves the partner chains in quite different positions (Supplementary Fig. S3). SSA buries about 780 Å^2^ of largely apolar surface of each chain at the interface; the residues involved represent a family specific insertion, conserved among SSA homologues but not found in other ricin family (R-type) lectins^26^. The BEL *β*-trefoil dimer interface has a similar surface area to that of SSA, but the interface is hydrophilic and association is driven by hydrogen bonding^15^.

### Sugar binding sites

The very high-resolution diffraction data allowed the GalNAc ligands to be placed unambiguously at three sites per polypeptide, related by the pseudo-symmetry of the protein sequence. The principal side-chain contacts made by MytiLec with GalNAc are all charged, with the ligand cradled by three histidines and two carboxyl groups (Fig. 4). Five hydrogen bonds form between the protein and each of the six ligand molecules (Fig. 4B). A HxDxH motif is found in each sub-domain (Supplementary Fig. S2), the first histidine (His 32, His 80 or His 124) lying against the sugar ligand but making no hydrogen bond with it. MytiLec and CGL are highly unusual among *β*-trefoil lectins in having histidine make this interaction, and not a larger aromatic side-chain. The aspartic acid and last histidine of the HxDxH motif make hydrogen bonds with O3 (Fig. 4B). The other side of the sugar lies against a conserved HPxGG motif found in each sub-domain, beginning at His 15, His 63 or His 107. The nitrogen atom of the first glycine residue makes a hydrogen bond with O6 of the ligand, which also hydrogen bonds to Glu 118, Asp 26 or Glu 74 in binding sites 1–3 respectively. The axially positioned O4 atom makes two hydrogen bonds to histidine residues, the first residue of the HPxGG motif and the last one of the HxDxH motif. D-glucose differs from D-galactose only in the position of this epimeric oxygen, so the structure implies strong selectivity for galactose over glucose. The C2-N-acetyl group of the ligand points away from the protein and makes no significant contact with it, indicating that galactose will bind as well as GalNAc with the same binding mode. (Arg 38 of one subunit does make a hydrogen bond to the acetyl of one ligand molecule, but the other five ligand molecules do not show this bond, which is presumably not strong.).

In order to observe any changes in the protein on ligand binding, both the apo and GalNAc complex structures were determined. Both the apo crystal and the co-crystal with ligand were cryo-protected immediately prior to flash-cooling by soaking in mother liquor containing 25% glycerol. At such high concentration glycerol is able to mimic the ligand binding, but not enough to displace GalNAc. The electron density map is shown in Supplementary Fig. S4. Comparing the main chain atoms of a single subunit, the apo and liganded forms show a rmsd of up to 0.35 Å, depending on which subunits are compared, indicating that the protein shows no movement on ligand binding. Crystal contacts in the different space-groups also do not apparently distort the protein significantly.

From their recent high resolution models of CGL complexes, Liao and colleagues report that the ligand binding modes of CGL and MytiLec are highly conserved, with only subtle differences between them^20^. They emphasise the importance of certain water molecules that mediate interactions between CGL and ligands, but the water structure of MytiLec bound to GalNAc shows differences among sites (even equivalent sites within the asymmetric unit), partly due to nearby crystal contacts, and offers little evidence of tightly held water molecules adding significantly to the affinity of binding. One surprising finding is that galactose is recognised as the *α*-anomer in all but one of the CGL ligand binding sites in the asymmetric unit, site 3 of chain A (PDB 5F8W)^20^, whereas the difference electron density map of the MytiLec-GalNAc complex shows no significant occupancy of the *β* anomer at any site. The anomeric hydroxyl group of the galactose *β* anomer binds to the carbonyl group of Gly 111 in CGL^20^, but this cannot explain the difference since site 3 is completely conserved between CGL and MytiLec, and an equivalent hydrogen bond is possible at each site (1–3) in both proteins. (Residue numbering in the CGL models is shifted by 1 relative to the MytiLec models). In the CGL-galactose crystal structure (PDB 5F8W), it is found that a neighbouring molecule comes close to site 3 of CGL, so that Arg 84 of this adjacent protein chain can accept a hydrogen bond at its carbonyl oxygen from the galactose *β* anomer, but not the *α*-anomer. Possibly this CGL crystal contact is responsible for the ligand conformation at this location, but clearly the *β* anomer of galactose or GalNAc is not excluded by steric clashes at any site, and the strong preference for the *α* anomer arises from other considerations. Although not found for each GalNAc molecule, a water-mediated hydrogen bond between O1 of the ligand and Asp 26 (or equivalent) may contribute to the anomeric selectivity of MytiLec, but as noted above the pattern of hydration is ambiguous.

**Figure 5:**
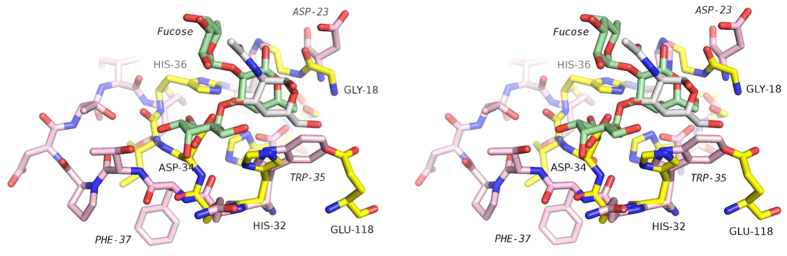
An overlap of liganded MOA and Mytilec monomers. Superposition was carried out with SSM^27^. The program selected 130 residues for overlap, with only 9.2% sequence identity, giving an rmsd of 1.7 Å. The carbon atoms of MytiLec and the GalNAc ligand are shown in yellow and white respectively. MOA and the blood group B trisaccharide, Gal*α*(1,3) [Fuc*α*(1,2)]Gal, are coloured with pink and pale green carbon atoms respectively^17^. The figure shows a close-up of site 1. Residue numbers for MOA are shown in an italic font. It can be seen that the non-reducing galactose residue of the trisaccharide overlaps the GalNAc bound to MytiLec, but Asp 34 of MytiLec clashes severely with the reducing Gal residue. Equivalent clashes are seen at each MytiLec binding site. Overall the binding sites of the two proteins are very different, with MOA having a much more extended surface loop, and Trp 35 (MOA) replacing His 32 (MytiLec). Behind Trp 35, it can be seen that His 15 of MytiLec is replaced by Asp 20 in MOA.

**Figure 6:**
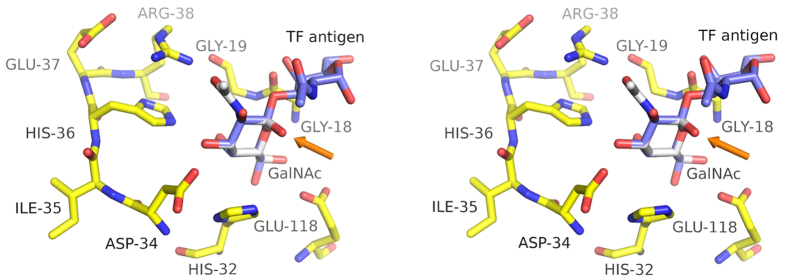
Structural basis of selectivity for *α*-linked substrates. This stereo view of GalNAc bound to MytiLec shows the carbon atoms of the ligand in white, and the anomeric oxygen atom is indicated by an orange arrow. TF-antigen, Gal*β*(1,3)GalNAc, overlapped onto the ligand is shown with carbon atoms coloured purple. Least-squares fitting matched the six carbon atoms of the galactose residue of TF-antigen, taken from PDB 4I4X, to equivalent atoms in the MytiLec-bound GalNAc. This causes strong clashes between the GG motif of the protein and the GalNAc residue of TF-antigen due to the *β*-linkage.

In order to understand how MytiLec might bind disaccharides or longer sugar chains selectively, SSM was used to overlap the protein with MOA carrying a branched trisaccharide, Gal*α*(1,3)[Fuc*α*(1,2)]Gal. The overall sequence identity between the two proteins is very low, but the backbone atoms fit closely, as indicated by the DALI score given above. Although fitting the protein backbone atoms places the reducing galactose residue of the MOA ligand over the GlcNAc bound to MytiLec, severe clashes can be seen with the non-reducing galactose residue (Fig. 5). The ligand binding site of MytiLec is too small and shallow for extensive contacts with oligosaccharide ligands, and the binding of melibiose (Gal*α*(1,6)Glc) suggests that only the non-reducing galactose residue is strongly recognised. The structural basis for the selectivity for *α*-linked saccharides can be explained by attempting to fit TF-antigen, Gal*β*(1,3)GalNAc*α*, to the MytiLec model (Fig. 6). The model of TF-antigen was taken from the high-resolution structure of BEL *β*-trefoil^15^. An in-house program called CFIT (JRHT, unpublished) was used to make least-squares superpositions of the carbon atoms of one or other residue of TF-antigen with the GalNAc bound to MytiLec. Fitting the N-acetyl-D-galactosamine residue of TF-antigen over the MytiLec ligand gave severe clashes with the aspartic acid of the HxDxH motif. Fitting the galactose residue of TF-antigen instead gave a better fit, but the extra residue showed strong steric clashes with the glycine-glycine dipeptide on the opposite side of the binding site (Fig. 6). The model suggests that MytiLec should be able to bind linear oligosaccharides with galactose or GalNAc at the non-reducing end, given an appropriate *α*-linkage to the next residue in the chain.

To confirm the binding specificities implied by the structural model, ITC was used to measure the affinity of MytiLec for different small sugar ligands, fitting the binding curve to a simple model with one type of independent site. GalNAc was found to bind with a *K*_*d*_ of 0.13 mM, and galactose with a *K*_*d*_ of 0.20 mM. A representative thermogram for GalNAc binding to MytiLec is shown in Supplementary Fig. S5, but the monomer mutant, MytiLec-F93DF94S, proved too insoluble for study by ITC. Glucose and derivatives were not found to bind MytiLec.

### Cytotoxicity and haemagglutination activity of the dimer and monomer forms of MytiLec

**Figure 7:**
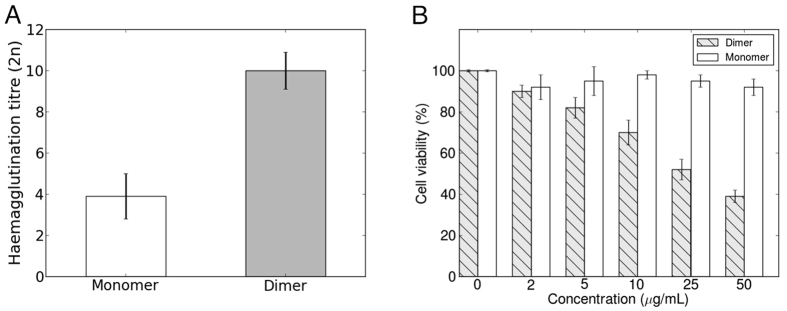
Haemagglutination activity and cell viability of recombinant MytiLec. (**A**) Haemagglutination titre of MytiLec. White and grey bars indicate haemagglutination titre of monomer MytiLec(F93DF94S) and native (dimeric) MytiLec, respectively. (**B**) Cell viability of Burkitt lymphoma Raji cells treated with MytiLec. Cell viability was determined using WST-8 assay. Raji cells were treated with various concentrations (0–50 *μ*g/ml) of MytiLec. White bars and grey shaded bars indicate the viability of cells treated with monomer or dimer MytiLec, respectively. Error bars show the standard error from three duplicates.

The presence of six ligand binding sites per protein molecule suggests that the multivalency may be functionally relevant. The monomer mutant MytiLec-F93DF94S possessed weak haemagglutination activity, around 10 times less than that of the dimer (Fig. 7A). Recombinant MytiLec (dimer) strongly reduces the viability of Burkitt’s lymphoma cells, with the same activity as native MytiLec, but MytiLec-F93DF94S (monomer) shows no cytotoxic activity at all (Fig. 7B). The three sugar binding sites of the monomer alone are apparently insufficient for the protein to kill suitable target cell lines, and the quaternary structure of the protein also plays a role in its cytotoxicity.

## Discussion

The ligand binding mode for MytiLec and the closely related CGL appears to be novel among *β*-trefoil lectins. Almost all such proteins have a phenylalanine or tyrosine side-chain lying against the apolar face of the ligand, a feature missing from MytiLec and replaced with the first histidine of the HxDxH motif. The sugar binding sites of MytiLec are well conserved in CGL, but not perfectly (Supplementary Fig. S6). The structure of the MytiLec-GalNAc complex suggested that MytiLec may have slightly higher ligand affinity than CGL due to the replacement of Glu 118 and Asp 26 with asparagine in CGL, at sites 1 and 2 respectively, weakening the hydrogen bond with O6 of the ligand (Fig. 4B). However, Liao and colleagues have recently reported experimental affinities of galactose for the three different sites on CGL using NMR, and shown that site 1 has a much higher affinity (*K*_*d*_ 178 *μ*M) than sites 2 or 3 (*K*_*d*_ 1288 *μ*M and 815 *μ*M, respectively); these data show no correlation with the presence of residues Asn 119, Asn 27 and Glu 75 at sites 1, 2 and 3 respectively of CGL. Their NMR experiments detected notable chemical shifts in only four site 1 residues (Ser 22, Ile 36, His 37 and Glu 38), whereas six or seven residues were affected at the other sites^20^. Possibly the weaker binding at sites 2 and 3 is due to slightly greater flexibility in the unliganded state, which incurs a free energy penalty to ligand binding, but such effects are not visible in the cryo-cooled MytiLec structures, despite the very high resolution, and further studies of mutants are required to determine the factors controlling the observed ligand affinity. Liao and colleagues also determined the structure of CGL with Gb3 allyl bound, and showed that the Gal1 residue makes the same interactions as galactose, but sites 1 and 2 have additional hydrogen bonds to Gal2 through Asn 119 and Asn 27 respectively^20^. They also suggest that Tyr 18 and Tyr 66 (but not the site 3 equivalent of Lys 110) contribute to Gb3 binding through water-mediated hydrogen bonds to Gal2. Biolayer interferometry showed Gb3 allyl binds CGL with a site-averaged *K*_*d*_ about three-times lower that that of galactose^20^. Both Tyr 18 and Tyr 66 residues are replaced in MytiLec, which shares Lys 110 with CGL, suggesting a lower affinity of MytiLec for Gb3, but the pattern of interactions is not entirely uniform at each site of the CGL-Gb3 complex (PDB 5F90). A comparison of MytiLec and CGL binding to Gb3 is shown in Supplementary Fig. S6. Kovalchuk and colleagues have built a homology model of CGL based on the structure of the MytiLec-GalNAc complex described here (PDB 3WMV), and used this model of ligand-bound CGL to mutate the binding sites individually^28^. Replacing the HPxGG motifs with AAxAG motifs at the first two ligand binding sites abolished haemagglutination and mucin binding, but eliminating the third binding site left residual activity, which is consistent with some role for Asn 27 and Asn 119 of CGL in binding the second sugar residue of cognate ligands. The MytiLec and CGL models show that the third binding site of each subunit lies on the same side of the dimer, whereas haemagglutination will presumably be promoted if attachment to one cell does not sterically hinder attachment to a second. Steric effects as well as multivalency and ligand affinity are apparently important influences on the cytotoxic activity of the protein.

The *β*-trefoil is a common fold, with many structures of widely divergent sequence known. Recently this domain family has been the subject of efforts by different groups to create symmetrical variants to help understand protein folding and evolution^24^,^29^,^30^. Lectins in this family are also known as R-type carbohydrate recognition domains (CRDs) after ricin, the toxin from castor beans. R-type CRDs occur widely among plants, animals and prokaryotes, either as part of larger polypeptides or as independent proteins, and over 8000 such sequences are known. Pierisin, from the larvae of the cabbage butterfly *Pieris rapae*, has three R-type lectin domains and an ADP-ribosyltransferase domain^31^, whereas MOA has two *α*-galactoside-binding R-type lectin domains and one serine protease domain. In both cases the non-lectin domain induces cell death^17^,^32^, but several *β*-trefoil lectins are known with no accessory domains that still have growth suppression activity against microorganisms or cancerous cells^33^,^34^. The sequence of MytiLec is sufficiently unusual for automated family assignment by Pfam^35^ or SMART^36^ to fail to recognize it as a ricin-type trefoil. The crystal structure described here reveals five amino acids in each subdomain that bind the hydroxyl-groups at the C3, C4 and C6 positions of galactose, and these binding sites (of MytiLec and its homologues CGL and MTL) appear to be quite unrelated to those of any other known lectin. A large majority of *β*-trefoil lectins bind to *β*-galactoside, but functional analysis shows that MytiLec prefers *α*-galactosides, and this ligand binding is required for the specific cytotoxic effects against certain cancer cell lines^1^. The molecular model directly explains this unusual ligand specificity, and why D-Gal and D-GalNAc are both recognized by MytiLec, since the protein does not interact strongly with groups at the C2 position of the sugar ligand. TF antigen is a well-known cancer cell marker saccharide that has galactose at the non-reducing end, like Gb3, but the MytiLec structure shows that it is unlikely to bind. MytiLec is somewhat unusual in binding ligand at each subdomain, and it forms a dimer in solution, giving a total of six ligand sites per protein. The fact that an artificial monomeric MytiLec showed weak haemagglutinating activity but no significant cytotoxicity against Raji cells indicates that the multivalent dimeric form of MytiLec is crucial for the cytotoxic effect. Recent studies have shown that MytiLec enters sensitive cells and initiates programmed cell death through multiple pathways including MAPK cascade and caspase activation^37^. Gb3 is a cell-surface marker found in several different cancer cell lines, which suggests that MytiLec and CGL may prove a useful basis for the development of new diagnostic agents or treatments for a variety of cancer types.

## Methods

### Cloning

A synthetic gene encoding MytiLec was designed with flanking NdeI and BamH1 restriction sites. Codon optimisation was performed using the back-translation tool from Entelechon, and the gene was synthesised by Operon. The initial reported residue of MytiLec is threonine. This was changed to alanine so that expression of the native sequence would result in efficient removal of the N-terminal methionine, but expression with an N-terminal histidine tag was exclusively used for protein production. The initiator methionine residue is numbered zero, in accordance with the UNIPROT entry B3EWR1. The designed DNA sequence was excised from the supplied plasmid DNA and inserted into appropriately cut pET28, using T4 DNA ligase (Wako) at room temperature for 1 h. The ligation mixture was used to transform *E*. *coli* DH5*α*, and pET28b-MytiLec was prepared from cultures using QIAprep (Qiagen). This vector directs expression of full-length MytiLec carrying a thrombin-cleavable hexa-histidine tag at the N-terminus. The final purified protein product, after tag removal, has a sequence beginning with GSHMATF.

### Expression and purification

pET28b-MytiLec was transformed into *E*. *coli* BL21(DE3) RILP, and cells were grown at 310 K with shaking in 6 litres of LB medium containing kanamycin and chloramphenicol (20 *μ*g/ml). When the O.D. 600 of the culture reached 0.6 ~ 0.7, MytiLec expression was induced by adding IPTG to a final concentration of 0.1 mM, and growth was continued overnight at 293 K. The cells were collected by centrifugation at 3000× *g* at 277 K for 30 min. The pellet was suspended in 100 mM Tris HCl pH 8.0/0.15 M NaCl/20 mM imidazole and then lysed by sonication on ice. The lysate was centrifuged at 38,000× *g* at 277 K for 50 min. The supernatant solution was loaded onto a 10 ml volume nickel-sepharose column (GE Healthcare) equilibrated with 100 mM Tris HCl pH 8.0, 0.15 M NaCl, 20 mM imidazole, and after washing, eluted with 20 mM Tris HCl pH 8.0, 500 mM imidazole, 150 mM NaCl. The major protein fractions were collected and digested with thrombin overnight at 277 K during dialysis into 20 mM Tris HCl pH 7.4/50 mM NaCl. The protease:MytiLec ratio was 1:200. The protein was re-loaded onto the washed nickel-sepharose column and eluted with 20 mM Tris HCl pH 7.4/50 mM NaCl. The pooled fractions containing MytiLec were dialysed into 20 mM Tris HCl pH 7.4/50 mM NaCl before loading onto an SP-sepharose column (GE) equilibrated with the same buffer, and eluted with a gradient to 1 M NaCl. The pooled protein fractions were concentrated to 10 mg/ml using Amicon centrifugal filter units (Millipore).

### Analytical Ultracentrifugation

Sedimentation velocity experiments were carried out using an Optima XL-I analytical ultracentrifuge (Beckman-Coulter) using an An-50 Ti rotor. Cells with a standard Epon two-channel centre-piece and sapphire windows were used. 400 *μ*l of the sample and 420 *μ*l of the reference solution (50 mM potassium phosphate pH 7.4 and 0.1 M NaCl) were loaded into the cell. The rotor was kept stationary at 293 K in the vacuum chamber for 1 h prior to each run for temperature equilibration. Absorbance at 280 nm scans were collected at 10 min. intervals during sedimentation at 50,000 rpm. The resulting scans were analysed using the continuous distribution *c*(*s*) analysis module in the program SEDFIT^38^. Frictional ratio (f/fo) was allowed to float during fitting. The *c*(*s*) distribution was converted into a molar mass distribution *c*(*M*). Partial specific volume of the protein, solvent density, and solvent viscosity were calculated from standard tables using the program SEDNTERP^39^.

### Crystallisation and structure determination

Crystallisation experiments were performed at 293 K using the hanging-drop vapor diffusion method. Crystals grew in 24% (w/v) PEG 4000, 20 mM sodium acetate, 0.1 M HEPES pH 7.5, 1% glycerol. Crystals were washed briefly in mother liquor containing 18.5% glycerol as cryo-protectant before being stored in liquid nitrogen. Data were collected at beam-line 1A of the Photon Factory, Tsukuba. Data were also collected from a crystal which had been soaked for 3 h in 0.5 mM K_2_PtCl_4_. A total of 250 images of 1° oscillation were collected for the native dataset, and 720 images for the derivative. Data processing and scaling were carried out with HKL2000 and SCALEPACK^40^. Two molecules were found in the asymmetric unit of the native structure. Eight platinum ions were located for the derivative, none with occupancy above 30%. Phases were calculated by SAD using PHENIX^41^, giving a mean FOM of 0.269. Model building was carried out with the Autobuild module^42^. Manual modifications were carried out with COOT^43^. Refinement was carried out with REFMAC^44^ and the CCP4 suite^45^. Non-crystallographic symmetry restraints and TLS group refinement were not applied. Isotropic temperature factors were refined with default isotropic restraints giving an R-factor around 15%. Finally anisotropic temperature factors were refined, dropping both the R-factor and free R-factor 2%. Water molecules were checked manually for steric clashes or unusually shaped electron density; several were fitted with partial occupancy. Water molecules were removed from the model if their refinement with anisotropic temperature factors proved unstable. Six glycerol molecules were identified by eye in the apo-MytiLec structure and refined with expected geometrical features. Figures were prepared with PYMOL^46^. Co-crystals of MytiLec were grown using 10 mg/ml protein with 5 mM N-acetyl-D-galactosamine. The reservoir solution contained 0.2 M potassium thiocyanate, 0.1 M bisTris-propane pH 8.5 and 20% PEG 3350. 25% glycerol was added to the reservoir solution for soaking crystals prior to cryo-cooling. Data were collected and processed similarly to the apo data-set. The complex structure was determined by molecular replacement from the apo model, and refined in a similar manner. Six molecules of N-acetyl-D-galactosamine were found bound to a protein dimer in the asymmetric unit of the complex structure, and all of them were refined as fully occupied. Data collection and refinement statistics are shown in Table 1.

### Creation of the Mytilec monomer mutant

MytiLec(F93DF94S) was produced by introducing both mutations simultaneously with a simple PCR procedure using Primestar polymerase. Overlapping 5′ and 3′ regions of the gene were amplified from the wild-type gene in pET28 using standard T7 promoter and T7 terminator primers, and two mutagenic primers: fw: CTATTCGCGATGGATTCTGACAATGATAACATTATACAC and rv: GTGTATAATGTTATCATTGTCAGAATCCATCGCGAATAG. The two reaction products were then combined and used as template for a third PCR reaction with only T7 primers present. The final reaction product was purified and cut with NdeI and BamH1 to ligate it into suitably cut pET28. Expression and purification of the mutant were carried out essentially as for the wild-type protein.

### Purification of native MytiLec

Native MytiLec was purified from the Mediterranean mussel *Mytilus galloprovincialis* using affinity chromatography by melibiosyl-agarose gel as previously reported^1^.

### Haemagglutination activity assays of dimeric and monomeric MytiLec

Haemagglutination assays were performed in 96-well U-shape plates as described previously^47^. 20 *μ*l of a 2-fold dilution of each recombinant MytiLec (native dimer or mutant monomer, 20 mg/ml starting concentration) in TBS was mixed with 20 *μ*l of a 1% suspension (with TBS; v/v) of fresh rabbit erythrocytes that was washed by saline. The plate was incubated at room temperature for 1 h, and the formation of a sheet (agglutination-positive) or dot (agglutination-negative) was observed and scored against the lectin titre.

### Cytotoxicity of recombinant MytiLec

Raji cells were maintained in RPMI 1640 medium supplemented with heat-inactivated fetal calf serum 10% (v/v), penicillin (100 IU/ml), and streptomycin (100 *μ*g/ml) at 310 K in an atmosphere of 95% air/5% CO_2_. Cytotoxic activity and cell growth were determined using Cell Counting Kit-8 containing WST-8 (Dojindo Molecular Technologies Inc., Kumamoto, Japan)^31^,^48^. Cells (2 × 10^4^, in 90 *μ*l solution) were seeded into a 96-well flat-bottom plate and treated with various concentrations of the recombinant MytiLec (native dimer or monomer mutant; 10 *μ*l of 0–50 *μ*g/ml) for 24 h at 310 K. The effect on cell growth was assayed by addition of WST-8 solution (10 *μ*l) to each well and incubation for 4 h at 310 K. The reduction in the proportion of living cells was assayed by measurement of absorbance at 450 nm (relative to reference absorbance at 600 nm) using the GLOMAX Multi Detection System (Promega, Madison, WI USA). Results of experiments are presented as the mean ± standard error. Differences in means were evaluated by two-tailed Student’s *t*-test with P values < 0.05.

### Isothermal titration calorimetry

ITC experiments were carried out with a MicroCal VP-ITC (Malvern). 20 *μ*M MytiLec (in 10 mM HEPES pH 7.4, 100 mM NaCl) was placed in the cell, and maintained at a temperature of 298 K. Ligand was dissolved in the same buffer to a final concentration between 5 mM and 10 mM. 22 injections of ligand, 10 *μ*l each, were made in total, allowing the baseline to stabilise between injections. The raw data were fitted to a single site model using the manufacturer’s software.

## Additional Information

**Accession codes:** The final models and structure factors are available from the Protein DataBank with codes 3 WMU and 3 WMV.

**How to cite this article**: Terada, D. *et al*. Crystal structure of MytiLec, a galactose-binding lectin from the mussel *Mytilus galloprovincialis* with cytotoxicity against certain cancer cell types. *Sci. Rep.* **6**, 28344; doi: 10.1038/srep28344 (2016).

## Supplementary Material

Supplementary Information

## Figures and Tables

**Table 1:** Crystallographic data statistics.

Data collection statistics			
Data-set	Apo	GalNAc	Pt
Space group	*P*2_1_	*C*2	*P*2_1_
Wavelength (Å)	1.1000	0.9800	1.0713
Unit cell (Å)	a = 57.7, b = 41.5	a = 80.3, b = 64.2	a = 57.3, b = 41.7
	c = 68.8, *β* = 108.5°	c = 74.3, *β* = 121.5°	c = 68.2, *β* = 108.1°
Resolution range (overall/outer shell)	50.0-1.10/1.12-1.10	28.6-1.049/1.088-1.05	50.0-1.79/1.82-1.79
Reflections (measured/unique)	422,688/120,340	650,349/147,442	133,713/28,579
Completeness (overall/outer shell, %)	96.0/89.6	98.6/97.9	98.4/97.1
^*a*^R_*merge*_ (overall/outer shell, %)	10.8/29.8	6.6/41.4	8.5/29.7
Multiplicity (overall)	3.5	4.4	4.7
Average *I*/*σ*(*I*) (overall/outer shell)	15.3/2.1	34.4/6.2	15.0/4.2
Refinement statistics			
	Apo (PDB 3 WMU)	GalNAc (PDB 3 WMV)	
Resolution range (Å)	27.48-1.10	28.65-1.05	
^*b*^R-factor/free R-factor (%)	13.7/16.3	13.7/15.5	
Rmsd bond lengths (Å)/angles (°)	0.024/2.23	0.024/2.15	
No. water molecules	341	299	
Average B factors (Å^2^) (protein/water/ligand)	10.0/24.2/-	8.6/20.9/12.3	
% residues with favoured Ramachandran angles	96.7	96.7	
% residues with acceptable Ramachandran angles	3.3	3.3	
% residues with outlier Ramachandran angles	0	0	

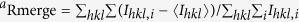
, where *I*_*hkl*,*i*_ is the intensity of an observation and 〈*I*_*hkl*_〉 is the mean value of all intensity observations for this reflection. Values in parentheses are for the highest resolution shell. ^*b*^R factor is 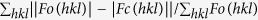, where *Fo* and *Fc* are the observed and calculated structure factor amplitudes, respectively. The free R factor was calculated with 5% of reflections omitted from the refinement.
