# Supplementary material for: Crystal structure of MytiLec, a galactose-binding lectin from the mussel Mytilus galloprovincialis with cytotoxicity against certain cancer cell types
Source: Sci Rep. 2016 Jun 20;6:28344. doi: 10.1038/srep28344 (PMC4913266; doi:10.1038/srep28344)
Supplement: Supplementary Information [file srep28344-s1.pdf]

Crystal structure of MytiLec, a galactose-binding lectin from the mussel *Mytilus galloprovincialis* with cytotoxicity against certain cancer cell types.

Daiki Terada[1], Fumihiro Kawai[1], Hiroki Noguchi[1], Satoru Unzai[1], Imtiaj Hasan[2,3], Yuki Fujii[4], Sam-Yong Park[1], Yasuhiro Ozeki[2], and Jeremy R. H. Tame[1,*]

[1]Graduate School of Medical Life Science, Yokohama City University, 1-7-29 Suehiro, Yokohama, Kanagawa 230-0045, Japan
[2]Laboratory of Glycobiology and Marine Biochemistry, Graduate School of NanoBio Sciences, Yokohama City University, 22-2, Seto, Yokohama, Kanagawa 236-0027, Japan
[3]Department of Biochemistry and Molecular Biology, Faculty of Science, University of Rajshahi, Rajshahi-6205, Bangladesh
[4]Department of Pharmacy, Graduate School of Pharmaceutical Science, Nagasaki International University, 2825-7 Huis Ten Bosch, Sasebo, Nagasaki 859-3298, Japan
[*]jtame@tsurumi.yokohama-cu.ac.jp

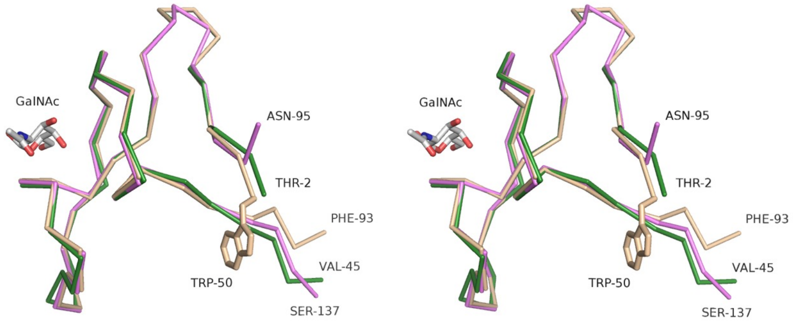

Figure S1. A stereo-view of the individual sub-domains of one MytiLec polypeptide chain overlaid by least-squares superposition of Cα carbon atoms. Sub-domain 1 (residues 2-45) is shown in green, sub-domain 2 (residues 50-93) is shown in cream, and sub-domain 3 (residues 95-137) is shown in pink. The single tryptophan residue of the protein (Trp 50) is found just after a loop region that is slightly longer than its counterpart between sub-domains 2 and 3. One molecule of GalNAc (shown as sticks with white carbon atoms) indicates the position of the ligand binding site relative to the protein fold.

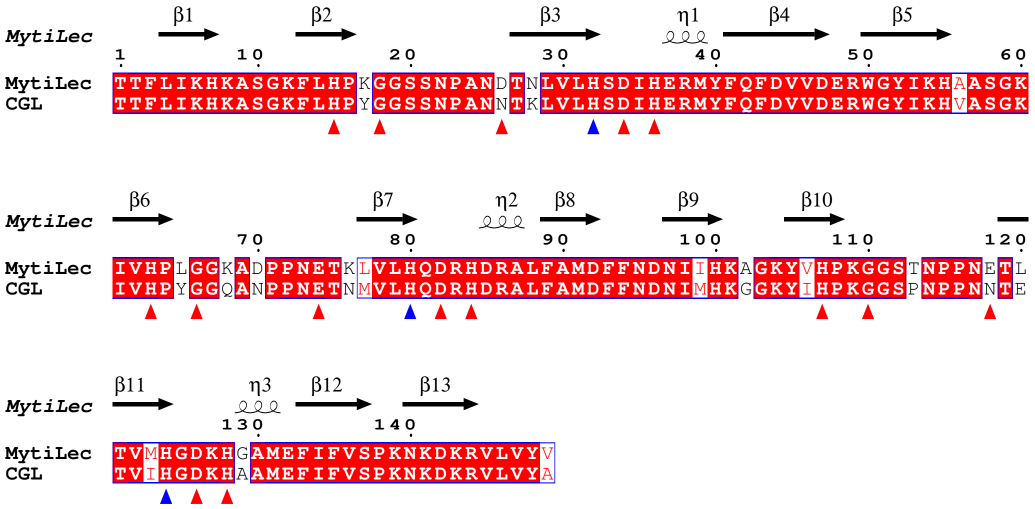

Figure S2. A sequence alignment of MytiLec and CGL, showing the secondary structure. Residues forming hydrogen bonds with the ligand are indicated with red triangles. The histidine making van der Waals contacts with the ligand is shown with a blue triangle. The galactose binding sites are highly similar between MytiLec and CGL, the main difference being residues Asp 26 and Glu 118, at binding sites 2 and 1 respectively. The replacement of the carboxyl-bearing side-chains with asparagine in CGL suggests that these binding sites will have lower ligand affinity than MytiLec. Glu 74 of binding site 3 is conserved between MytiLec and CGL.

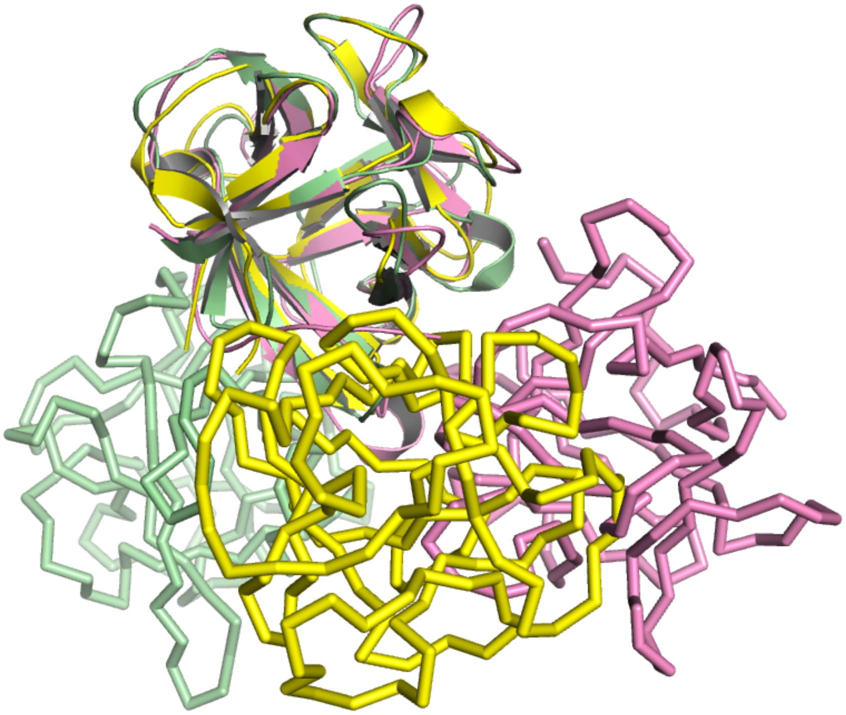

Figure S3. An overlay of the MytiLec dimer with dimers of SSA and BEL β-trefoil. One subunit of each protein (shown with β-strands as ribbons) was fitted by least-squares overlay using SSM. The other subunit is shown as a ribbon, indicating the relative position of the subunits in each protein is very different. MytiLec is shown in yellow, SSA in green, and BEL β-trefoil in pink.

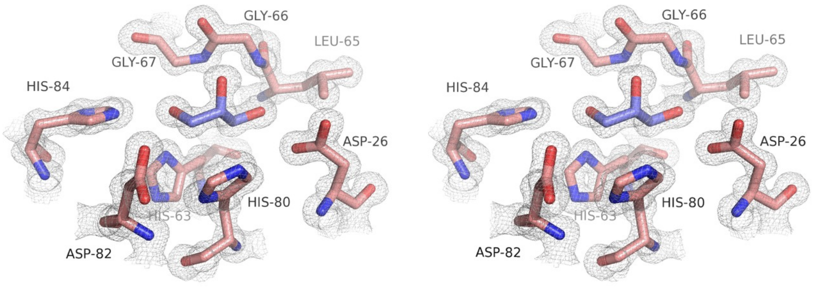

Figure S4. A stereo figure of the 2mFo-DFc electron density map (1 σ) of apo-MytiLec covering a glycerol molecule. The carbon atoms of the protein are shown in brown, and of the glycerol in purple. Glycerol mimics the sugar ring, but the central hydroxyl group lies where the ring oxygen atom of galactose is found, and consequently has no hydrogen bonding partner on the protein. Glycerol lacks an equivalent to the equatorial oxygen O3 of the sugar, and so forms no interactions with the DxH residues found in each sub-domain.

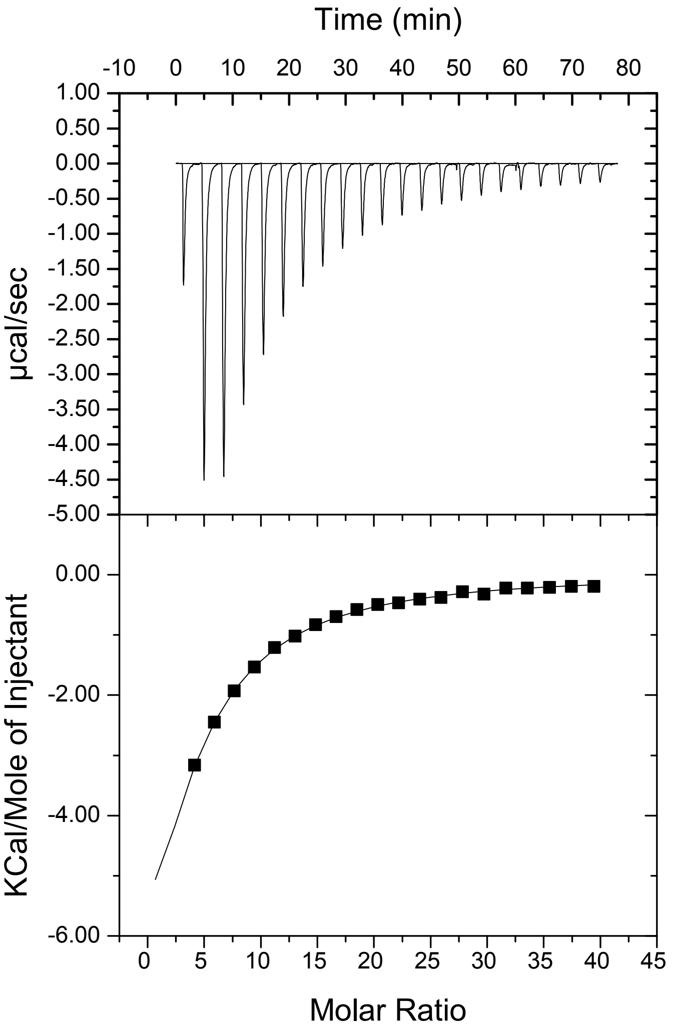

Figure S5. Binding of GalNAc to MytiLec determined by isothermal titration calorimetry. Ligand concentration was 5 mM. The upper panel shows the raw thermogram, and the lower panel shows the fitted data. The best-fit K_d was 0.13 mM, with a stoichiometry of 2.6 ± 0.4 ligands per protein monomer. No initial plateau region is seen in the early injections due to the relatively high ligand concentration used to give sufficient heat response from the weak binding. Various protein-ligand ratios were tested, and the optimal combination is shown.

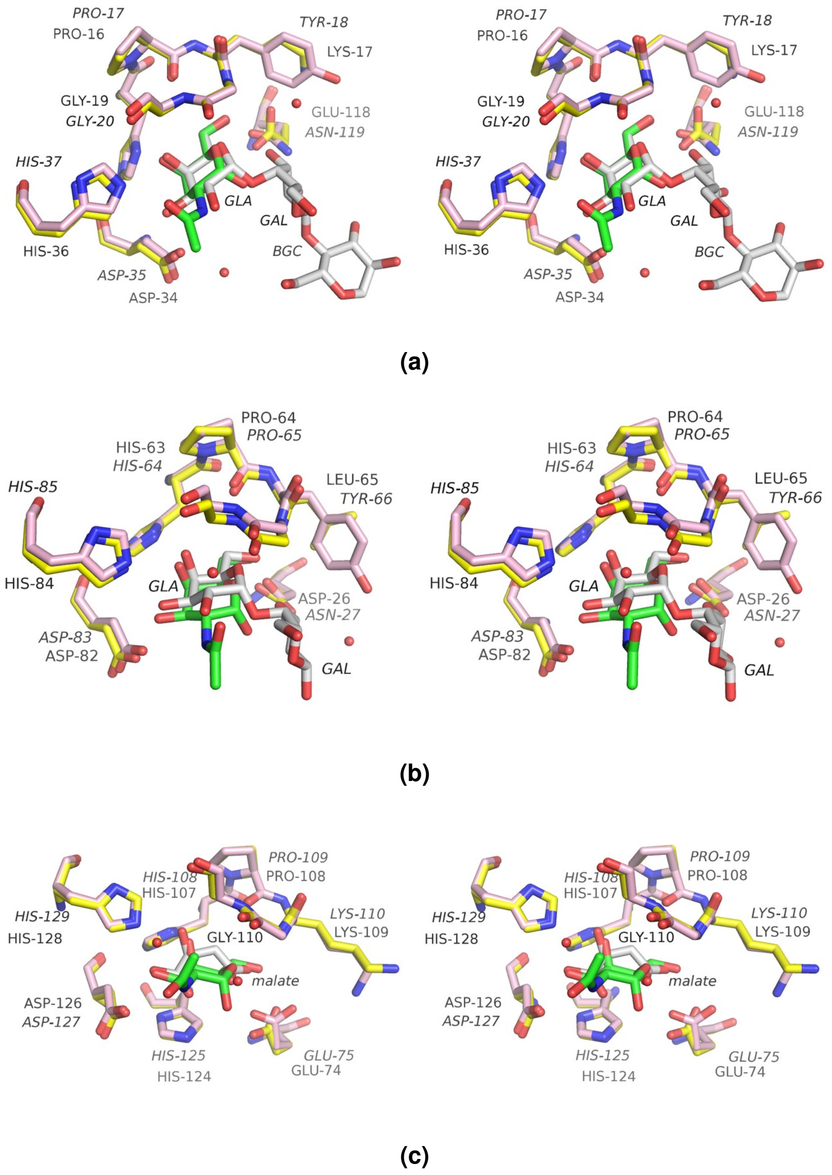

Figure S6. Overlay of the MytiLec-GalNAc and CGL-Gb3 allyl complexes. PDB 3WMV was overlaid onto PDB 5F90 by SSM as a dimer, and the three binding sites of chain A are shown. Labels in italics refer to the CGL complex. MytiLec carbon atoms are coloured yellow, GalNAc carbon atoms are coloured green, CGL carbon atoms are coloured pink, and the carbon atoms of CGL ligands are coloured white. Nearby water molecules of the CGL complex are shown as red spheres. Water is not shown for the MytiLec complex. (a) The non-reducing galactose of Gb3 matches closely the position of GalNAc, but the second and third residues of Gb3 allyl point away from the protein and make little contact with it. (b) Site 2. Only two residues of the ligand sugar were fitted to CGL. (c) Site 3. A malate ion was fitted in the binding site of CGL.

Buried area by residue (PDB 3WMV)		
Type	Number	Buried area
Chain A		
VAL	45	11.6
ASP	47	27.4
GLU	48	96.0
ARG	49	86.7
ARG	90	5.5
MET	91	3.5
ASP	92	29.2
PHE	93	73.4
PHE	94	133.3
ASN	95	39.5
ASN	96	0.2
ASN	99	4.3
ASN	135	0.2
LYS	143	35.1
ARG	144	47.9
VAL	145	9.8
LEU	146	66.7
TYR	148	52.0
Total		722.3
Chain B		
VAL	45	11.9
ASP	47	34.2
GLU	48	96.2
ARG	49	86.8
ARG	90	5.0
MET	91	3.2
ASP	92	29.6
PHE	93	73.1
PHE	94	132.6
ASN	95	39.8
ASN	96	0.2
ASN	99	4.5
LYS	143	29.4
ARG	144	44.1
VAL	145	9.9
LEU	146	66.1
TYR	148	52.6
VAL	149	33.2
Total		752.4

Table S1. Buried surface area at the dimer interface of MytiLec. Buried surface was determined per atom using AREAIMOL from the CCP4 suite, and then summed by residue. Water molecules were ignored for the calculation. Roughly 1470 $Å^2$ of surface area is buried at the interface by dimer formation. The essential contribution of Phe 94 to dimerisation is evident.

Inter-subunit distances for PDB 3WMV						
Atom 1			Atom 2			Distance (Å)
A	143(LYS)	NZ	B	47(ASP)	CG	3.42
			B	47(ASP)	OD1	2.82
A	347(HOH)	O	B	47(ASP)	OD1	3.23
A	143(LYS)	NZ	B	47(ASP)	OD2	3.50
A	93(PHE)	O	B	49(ARG)	CZ	3.34
			B	49(ARG)	NH1	2.87
A	144(ARG)	NH1	B	49(ARG)	NH1	3.26
A	93(PHE)	O	B	49(ARG)	NH2	3.24
A	310(HOH)	O	B	49(ARG)	NH2	3.03
A	94(PHE)	CG	B	92(ASP)	CG	3.35
			B	92(ASP)	OD1	3.44
A	94(PHE)	C	B	92(ASP)	OD2	3.39
A	49(ARG)	CZ	B	93(PHE)	O	3.34
A	49(ARG)	NH1	B	93(PHE)	O	2.88
A	49(ARG)	NH2	B	93(PHE)	O	3.20
A	92(ASP)	OD2	B	94(PHE)	C	3.44
A	92(ASP)	CG	B	94(PHE)	CG	3.32
A	92(ASP)	OD1	B	94(PHE)	CG	3.40
A	92(ASP)	CG	B	94(PHE)	CD1	3.50
A	95(ASN)	ND2	B	95(ASN)	CG	3.50
A	95(ASN)	OD1	B	95(ASN)	OD1	3.38
A	95(ASN)	ND2	B	95(ASN)	OD1	3.01
A	416(HOH)	O	B	95(ASN)	OD1	2.95
A	95(ASN)	OD1	B	95(ASN)	ND2	3.07
A	95(ASN)	ND2	B	95(ASN)	ND2	3.39
A	47(ASP)	OD1	B	143(LYS)	NZ	3.06
A	49(ARG)	NH1	B	144(ARG)	NH1	3.34
A	305(HOH)	O	B	145(VAL)	O	2.74
A	149(VAL)	CG1	B	148(TYR)	O	2.85
A	148(TYR)	O	B	149(VAL)	CG1	2.94
A	305(HOH)	O	B	301(HOH)	O	2.81
A	145(VAL)	O	B	305(HOH)	O	2.77
A	93(PHE)	C	B	305(HOH)	O	3.46
A	303(HOH)	O	B	305(HOH)	O	2.80
A	308(HOH)	O	B	305(HOH)	O	2.84
A	305(HOH)	O	B	306(HOH)	O	2.83
A	49(ARG)	NH2	B	314(HOH)	O	3.04
A	388(HOH)	O	B	323(HOH)	O	2.87
A	342(HOH)	O	B	330(HOH)	O	2.67
A	334(HOH)	O	B	339(HOH)	O	2.74
A	347(HOH)	O	B	362(HOH)	O	2.78
A	386(HOH)	O	B	369(HOH)	O	2.89
A	442(HOH)	O	B	400(HOH)	O	3.11
A	318(HOH)	O	B	400(HOH)	O	2.88

Table S2. Distances no greater than 3.5 Å between the two subunits of the MytiLec dimer, including nearby ordered water molecules.
